# Electrospun 3D Structured Carbon Current Collector for Li/S Batteries

**DOI:** 10.3390/nano10040745

**Published:** 2020-04-14

**Authors:** Sandugash Kalybekkyzy, Almagul Mentbayeva, Yerkezhan Yerkinbekova, Nurzhan Baikalov, Memet Vezir Kahraman, Zhumabay Bakenov

**Affiliations:** 1 National Laboratory Astana, Nazarbayev University, Institute of Batteries, Nur-Sultan 010000, Kazakhstan; sandugash.kalybekkyzy@nu.edu.kz (S.K.); yerkezhan.yerkinbekova@nu.edu.kz (Y.Y.); zbakenov@nu.edu.kz (Z.B.); 2 School of Engineering and Digital Sciences, Nazarbayev University, Nur-Sultan 010000, Kazakhstan; nurzhan.baikalov@nu.edu.kz; 3 Department of Chemistry, Marmara University, Istanbul 34722, Turkey; mvezir@marmara.edu.tr

**Keywords:** carbon nanofibers, lithium-sulfur battery, electrospinning method, electrode capacity, current collector

## Abstract

Light weight carbon nanofibers (CNF) fabricated by a simple electrospinning method and used as a 3D structured current collector for a sulfur cathode. Along with a light weight, this 3D current collector allowed us to accommodate a higher amount of sulfur composite, which led to a remarkable increase of the electrode capacity from 200 to 500 mAh per 1 g of the electrode including the mass of the current collector. Varying the electrospinning solution concentration enabled obtaining carbonized nanofibers of uniform structure and controllable diameter from several hundred nanometers to several micrometers. The electrochemical performance of the cathode deposited on carbonized PAN nanofibers at 800 °C was investigated. An initial specific capacity of 1620 mAh g^−1^ was achieved with a carbonized PAN nanofiber (cPAN) current collector. It exhibited stable cycling over 100 cycles maintaining a reversible capacity of 1104 mAh g^−1^ at the 100th cycle, while the same composite on the Al foil delivered only 872 mAh g^−1^. At the same time, 3D structured CNFs with a highly developed surface have a very low areal density of 0.85 mg cm^−2^ (thickness of ~25 µm), which is lower for almost ten times than the commercial Al current collector with the same thickness (7.33 mg cm^−2^).

## 1. Introduction

Since the successful launch in the 1990s, the batteries based on lithium intercalation electrochemistry have dominated the market for more than two decades due to their relatively high operating potentials and long cycle life [1,2]. Li-ion batteries (LIBs) have become one of the most popular battery technologies in portable energy storage and electric vehicles due to their high energy and power density, high coulombic efficiency, and low rate of self-discharge. Conventional LIBs are composed of positive and negative electrodes that are electrically insulated by a porous polymer membrane wetted in an organic electrolyte with containing lithium salt. The electrode materials are usually deposited onto the current collectors. Usually, Al foil is used for the positive and Cu foil for the negative electrodes [3]. The electrochemical potential difference between the electrodes during the charge process moves the Li^+^ from the positive to the negative electrode through the electrolyte. The reverse process occurs during discharge [4,5]. In this process, a current collector significantly affects the battery performance. The current collector is an essential component to hold the electrode material, ensure good current conduction and collect the accumulated electrical energy from the electrode [6]. Two-dimensional (2D) Al foil is the most commonly used current collectors for cathodes in LIBs with low cost and mature manufacturing. However, the instability of Al metal in organic electrolytes is still a challenge hindering long-cycle sustainability and electrical conductivity [7]. In order to improve the electrical conductivity of the electrodes, various types of current collectors including nickel, stainless steel and carbon in the forms of thin foil, mesh and foam were developed [8,9,10,11,12]. Degradation upon a long-term operation, heavyweight, and weak adhesion of electrode material are still issues to be addressed regarding the current collectors [13,14]. Therefore, it is crucial to develop lightweight, chemically stable, mechanically durable current collectors with good adhesion of active materials for the use in next-generation LIBs.

Carbon materials are a promising alternative to conventional metal-based current collectors due to their good chemical/electrochemical stability and low density, which reduces the total weight of a cell and increases its overall gravimetric energy density. A variety of carbon current collectors such as carbon paper, graphite sheets, three-dimensional (3D) carbon foam, carbon fiber mats with high specific surface areas have been applied to improve cycle life and gravimetric energy density of electrode materials [15,16,17,18,19,20]. Particularly, carbon fibers (CFs) have many advantages like high conductivity, structural stability and large surface area to accommodate more electrode materials, which makes them attractive to use in energy storage devices [21,22]. Use of CFs with interconnected 3D void spaces as 3D structured current collector allows one to incorporate electrode materials into its structure and hold more active material per specific area. In addition, CFs provide higher interfacial interaction compared with 2D current collectors, where active material interacts only with its surface [23,24,25]. Beside this, CFs are cheaper than metallic 3D current collectors. Previously, H. Lu et al. successfully fabricated flexible, mechanical and chemically stable Li_4_Ti_5_O_12_ electrodes using commercial CFs as a current collector [26]. Additionally, Y. Zhang compared Ni foam and CF cloth as 3D current collectors for lithium–sulfur (Li/S) batteries. The cell with CF current collector exhibited enhanced electrochemical performance maintaining 1278 mAh g^−1^ initial capacity and stable cycle performance compared with the cell with Ni foam current collector offering only 750 mAh g^−1^ initially [27]. Moreover, there are several successful works on 3D carbon current collectors for both anode and cathode electrode materials [7,12,28,29]. H.-J. Peng et al. reported improved cyclability within sulfur composite cathode using a 3D current collector made of commercial carbon nanotubes (CNT), which offered a 1109 mAh g^−1^ initial capacity [6]. CNTs are an efficient sulfur host material and were able to act a current collector at the same time. However, the high cost and large surface area requesting a large amount of electrolyte limit their practical application. In a number of studies, commercial CFs with the diameters of more than 5 µm were investigated [11,26]. The larger diameters results in a higher electrical resistivity of the carbon fibers [11]. Additionally, it results in increased weight and decreased specific surface area of a current collector. Fabrication of CFs with the diameters less than 2 µm could be more beneficial from this point of view.

Electrospinning technology enables production of homogenous, continuous polymer fibers with the diameters in the nanometer range from polymer solutions in high electric fields [30]. This method is a promising way of CF production for the development of high performance 3D current collectors compared to conventional mechanical spinning techniques [31,32,33]. Electrospinning is one of the simplest and cost-effective techniques to produce carbon nanofibers (CNFs) [34]. Furthermore, it is easy to control/manage the diameter, morphology and density/structure of fibers by adjusting spinning parameters like polymer concentration, applied voltage, speed of rotation of drum and distance between nozzle and collector. Recently, Wu et al. developed CNF based current collector by the electrospinning method for Li^+^ ion deposition with a diameter of fibers around 250 nm [35]. The dendrite-free morphology and improved electrochemical performance were achieved.

Herein, we present the fabrication and investigation of electrospun carbon nanofibers from a polymer precursor with different diameters and void sizes, and their application as a 3D current collector for a sulfur composite electrode. It is well known that polyacrylonitrile (PAN) is the most popular precursor for CNFs with a high carbon yield (more than 50%) and excellent mechanical properties (up to 900 GPa in modulus) and thermal stability [36,37]. Carbonized polyacrylonitrile nanofibers (denoted as cPAN) were prepared from PAN solutions with concentrations of 10, 12 and 14 wt % in dimethylformamide (DMF) by the electrospinning method. The areal density of cPAN mat (obtained from 12 wt % PAN solution) was 0.85 mg cm^−2^ with a thickness of ~25 µm, while a commercial Al current collector with the same thickness weighs 7.33 mg cm^−2^. The ultralight and porous cPAN was able to accommodate a three times heavier amount of a sulfur composite cathode than its own weight, which results in sulfur mass loading of around 1.2 mg cm^−2^. The electrode delivered the initial specific capacity as high as 500 mAh g^−1^ based on the mass of the whole electrode.

## 2. Materials and Methods

Polyacrylonitrile (M_w_ 150,000, J&K Scientific Ltd., Beijing, China), sulfur (98% purity, GOST 127.1, Tengizchevroil, Kazakhstan), multiwalled carbon nanotubes (CNT, >95% purity, OD: 10–20 nm, US Research Nanomaterials, Inc. Inc., Houston, TX, USA), *N*,*N*-Dimethylformamide (DMF, 99% purity, Sigma-Aldrich, Germany), acetylene black (AB, MTI Co., Richmond, CA, USA), *N*-methyl-2-pyrrolidone (NMP, >99.5% purity, Sigma-Aldrich, Netherlands), polyvinylidene fluoride (PVdF, Kynar, HSV900, Richmond, CA, USA) and commercial CFs were used as received without further purification.

### 2.1. Fabrication of Carbon Nanofibers

Carbon nanofibers were fabricated by an electrospinning apparatus (Inovenso Ltd., Ne200, Istanbul, Turkey). Homogenous spinning solutions of PAN with the concentrations of 10, 12 and 14 wt % in DMF were prepared by stirring overnight and abbreviated as PAN10, PAN12 and PAN14, respectively. The polymer solutions were spun at room temperature for 1 h in air with the applied potential of 17 kV and a solution flow rate of 1 mL h^−1^. The distance between the rotating cylinder/drum and the tip of the capillary was 13 cm. Nanofibers were collected on an aluminum foil with a cylinder rotational speed of 100 rpm. The stabilization and carbonization of obtained PAN nanofibers were conducted in a tubular furnace (Across International, STF1200, Berkeley Heights, NJ, USA). A constant flow of air was maintained through the furnace during the stabilization. First, PAN nanofibers were stabilized by heating with the rate of 5 °C/min from room temperature to 280 °C and held at 280 °C for 1 h to complete the stabilization. The stabilized PAN nanofibers were easily removed from the foil and carbonized in argon at temperatures from 600 to 800 °C for 4 h with the heating rate of 10 °C min^−1^. The mass per unit area of the carbon nanofiber was about 0.85 mg cm^−2^.

### 2.2. S/DPAN/CNT Cathode at a Carbon Nanofiber Current Collector

Sulfur/dehydrogenated polyacrilonitrile/multiwalled carbon nanotube (S/DPAN/CNT) composite was synthesized as previously reported by our group [38]. First, S with PAN in a weight ratio of 75:25 and CNT (2 wt % of total mass) was manually grinded. Further, the mixture was heat treated in a tubular furnace at 300 °C for 4 h in argon to form the S/DPAN/CNT composite. The slurry was prepared by mixing 80 wt % S/DPAN/CNT composite, 10 wt % AB and 10 wt % PVDF in NMP. The resulting slurry of S/DPAN/CNT was applied onto electrospun prepared CNFs by vacuum infiltration technique and dried in a vacuum oven at 60 °C for 12 h.

For comparison, a conventional S/DPAN/CNT cathode on commercial CF and Al foil (thickness was ~15 µm, areal density was ~4.9 mg cm^−2^) was also prepared and tested under similar conditions. In this case, 80 wt % of S/DPAN/CNT composite, 10 wt % AB and 10 wt % PVdF were dispersed in NMP. The prepared slurry was casted on Al foil and commercial CF by the doctor blade technique. Specific capacity and current density were calculated based on the weight of sulfur in the electrode.

### 2.3. Structure Characterization and Electrochemical Measurements

The morphologies of the samples were studied by field-emission scanning electron microscopy, and energy-dispersive spectroscopy (SEM/EDS, FESEM, JEOL JSM-7500F, ZEISS Crossbeam 540, Jena, Germany). The structural properties of the samples were characterized by X-ray diffraction (XRD, Rigaku SmartLab, Japan) and Raman spectroscopy (HORIBA Scientific, France). The sulfur content in the composites was determined using a CHNS analyzer (Vario Micro Cube, Elementar, Germany).

S/DPAN/CNT composite cathodes incorporated with cPAN CNF current collector were used as working electrodes after vacuum drying at 60 °C overnight. The areal mass loading of sulfur for all samples was 1.2 ± 0.2 mg cm^−2^. The coin-type cells (CR2032) were assembled in an argon filled glovebox (MasterLab, MBraun, Germany) using lithium foil as both the negative and reference electrode, polypropylene membrane as a separator (Celgrad 2400) and lithium hexafluorophosphate (LiPF_6_) in ethylene carbonate/dimethylcarbonate/diethylenecarbonate (EC:DMC:DEC, volume ratio of 1:1:1, Targray) as an electrolyte. Cyclic voltammetry (CV) was conducted over a potential range from 1 to 3 V vs. Li^+^/Li at a scan rate of 0.1 mV s^−1^ (VMP-3 potentiostat/galvanostat, Bio-Logic Instruments, France). Galvanostatic charge-discharge was carried out on a multichannel battery tester (BT-2000, Arbin Instruments Inc., TX, USA) within the voltage range of 1.0–3.0 V (vs. Li^+^/Li). All the electrochemical measurements were carried out at room temperature.

## 3. Results and Discussion

**Figure 1:**
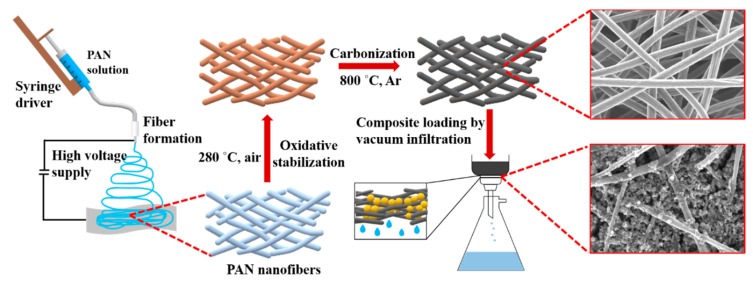
Scheme of carbon nanofiber fabrication and sulfur-based cathode preparation.

**Figure 2:**
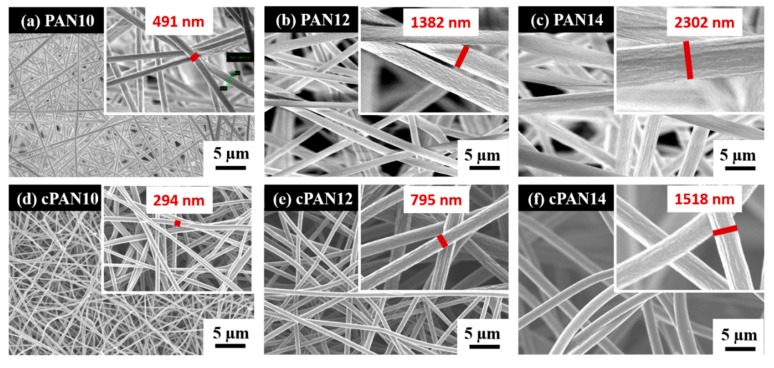
SEM images of (**a**) PAN10, (**b**) PAN12, (**c**) PAN14 and carbonized PAN nanofibers at 800 °C (**d**) cPAN10, (**e**) cPAN12 and (**f**) cPAN14.

Schematic representation of the CNF preparation and loading with S/DPAN/CNT cathode is shown in Figure 1. As illustrated, after electrospinning of PAN solution of different concentrations, obtained nanofibers were stabilized in air (Figure 1). Upon the stabilization process, PAN interacts with oxygen and forms cyclized macromolecules, which keep the fiber morphology unchanged during carbonization at high temperatures [39]. The carbonization of PAN nanofibers (NFs) was carried out within the temperature range from 600 to 800 °C in argon. Afterward, the structure and morphology of carbonized PAN (cPAN) NFs were characterized. The SEM images presented in Figure 2 show the morphology of the PAN and cPAN NFs derived from 10, 12 and 14 wt % PAN solutions and carbonized at 800 °C. The SEM images of the stabilized, and carbonized at 600 °C and 700 °C NFs are shown in Supplementary Information, Figure S1. All PAN nanofibers had uniform structure without any beads, and the fibers diameter for PAN10, PAN12 and PAN14 were approximately ~500 nm, ~1200 nm, and ~2400 nm, respectively. The morphologies of the cPAN NFs were similar to those of the precursor electrospun nanofibers (PAN) except for the differences in their diameters. The average diameter of the cPAN nanofibers was reduced almost by half of the precursor PAN nanofiber diameters, as summarized in Table S1. The change in diameter is due to the decomposition and evolution of gases like H_2_O, HCN, and N_2_ during carbonization process [40]. The void size between the fibers increased pursuant to SEM images. Considering the fiber diameter of ~800 nm and void size up to 1.5 µm, cPAN12 could be effectively used as current collector for sulfur composite cathode. The diameter and void size of cPAN10 were too small compared to S/DPAN/CNT composite material, which had a particle size in a range of 0.3–1.5 µm (Table S1). On the contrary, the cPAN14 fibers diameter was around 1.5 µm, and void size was around 3 µm, which complicated holding the active material densely packed in its structure and decreased electrical conductivity as well.

**Figure 3:**
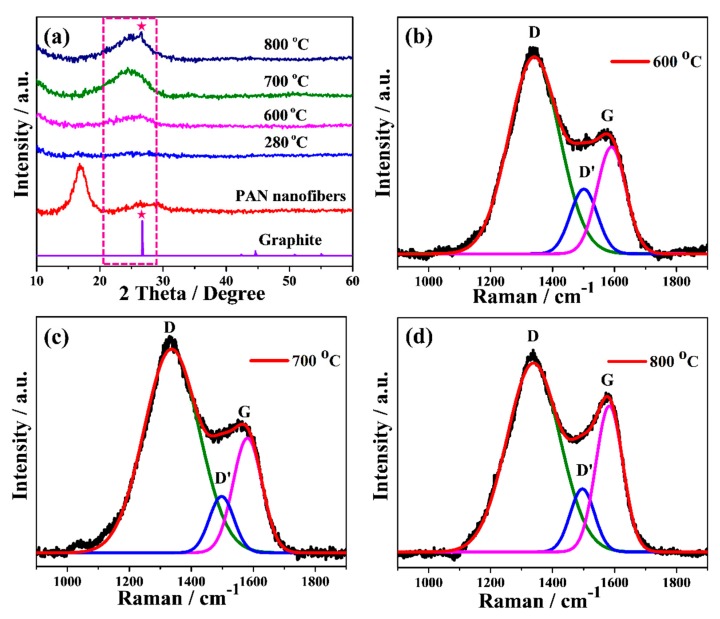
(**a**) XRD patterns and (**b**–**d**) Raman spectrum with the corresponding curve fitted bands of stabilized and carbonized PAN12 precursor nanofibers at different temperatures.

Figure 3a shows the XRD patterns of precursor PAN, stabilized and carbonized cPAN12 at different temperature. The electrospun PAN nanofiber showed a strong characteristic diffraction peak centered at 2*θ* ~17° and a broad amorphous scattering peak near 2*θ* ~27° [33]. However, these two peaks disappeared in the case of stabilized fibers, which indicates destruction of the PAN structure and its transformation into aromatic ring/ladder structures during the stabilization [41]. This indicates that stabilization of PAN NFs was sufficiently accomplished within 1 h of heating [42]. It should be noted that the stabilization process of conventional PAN took more than 1 h because of a limited heat diffusion rate in microsized fibers, therefore the process would be slower than for nanosized PAN [43]. Further, the effect of temperature was investigated through analysis of the XRD patterns of carbonized PAN NFs at different temperatures. The cPAN NFs at 600 °C showed a broad amorphous peak centered near 2*θ* ~25°. This peak could be related to the (002) c-axis disordered graphite-like material due to the stacking structure of aromatic layer (graphitic layer) [44]. With the increase of heating temperature, the intensity and sharpness of this peak gradually raised, suggesting the aromatic layers growth and an increased ordering between the graphitic layers [45]. It is known that the degree of graphitization rises with temperature [46,47], which is also confirmed by the sharp peak appeared at ~26.8° attributed to the (002) reflection of hexagonal graphite at carbonization temperature 800 °C. This can also be observed from the results of Raman spectroscopy, which is another effective tool for micro structural analysis of carbon based materials [48]. The Raman spectra of cPAN CNFs with the final carbonization temperatures of 600, 700 and 800 °C are presented in Figure 3b–d. For carbonized NFs it consists of three dominant peaks assigned to D, D’ and G bands, as shown in curve-fitting of the peaks of Raman spectra. The D band around 1340 cm^−1^ represents the disordered graphitic structure of the CNF and also signifies the sp^3^ content present in cPAN NFs [49]. The small and broad D’ peak at 1500 cm^−1^ also signifies the presence of defects, but it is due to amorphous sp^2^ bonded carbon [50]. The intensity of the D peak is higher compared to D’ in all samples, which shows the presence of more defects due to the amorphous sp^3^ than amorphous sp^2^. The G band around 1590 cm^−1^ indicates the ordered graphitic structure of the samples, and a broad shape of the peak indicates the small size of the CNFs crystallites [51]. However, it is noteworthy that with an increase in heating temperature intensity of G band in comparison with D band increased significantly, which shows an improvement in ordering graphitic layers of CNFs. The ordered structure of carbonaceous materials greatly affected the electrical conductivity, therefore the cPAN12 that carbonized at 800 °C were appropriate CNFs for application as current collector according to fiber diameter/void size as well, as mentioned above. Samples were not treated at temperatures above 800 °C since they became brittle and fragile.

**Figure 4:**
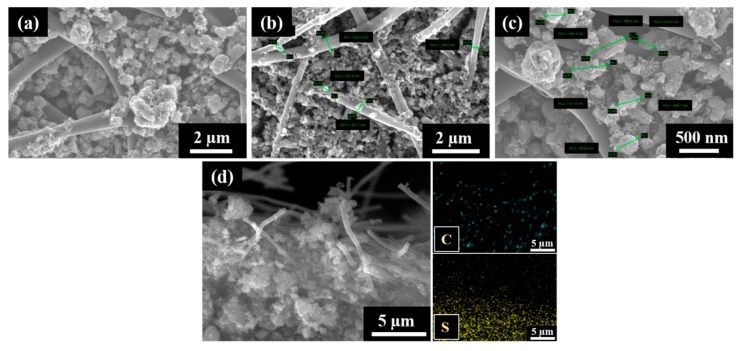
(**a**) SEM images of the sulfur/dehydrogenated polyacrilonitrile/carbon nanotube (S/DPAN/CNT) composite cathode on the carbonized polyacrylonitrile nanofibers (cPAN) carbon nanofiber (CNF) current collector, (**b**,**c**) cross-section views and (**d**) SEM/EDS mapping showing the distribution of S and C in the cross-section view of the electrode.

The S/DPAN/CNT composite slurry was prepared and applied into the cPAN12 NFs current collector. The slurry easily had penetrated into cPAN12 NFs by vacuum infiltration. The SEM images in Figure 4 display the uniform distribution of the composite cathode within the nanofibers. From the cross-section images in Figure 4b,c, it can be seen that S/DPAN/CNT composite penetrated deeply into the free spaces in internal CNF voids. The composite with the particle sizes less than 1.5 µm could easily penetrate into the current collector pores and be held well in its structure. Additionally, the distribution of composite material within the fibers was confirmed by SEM/EDS analysis. Figure 4d shows the cross-section image with the mapping of sulfur and carbon elements, which were penetrated and filled the void (free space) of nanofibers.

**Figure 5:**
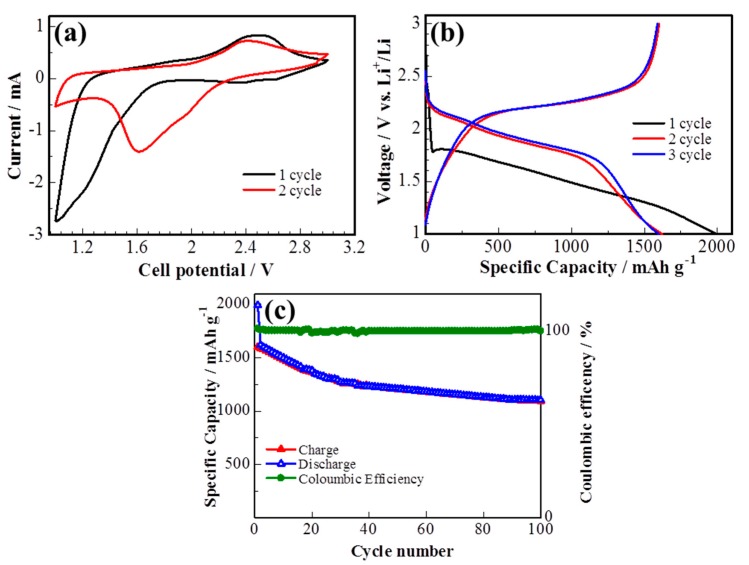
(**a**) Cyclic voltammetry (CV) curve, (**b**) potential profile and (**c**) cycle performance of the sulfur composite on cPAN CNFs at 0.1 C.

The electrochemical performance of the S/DPAN/CNT cathode on the 3D cPAN NF current collector was investigated in lithium half-cells by cyclic voltammetry (CV) and galvanostatic discharge/charge. Figure 5a shows the CV curve of the S/DPAN/CNT cathode during lithiation/delithiation processes within a potential range of 1.00–3.00 V at a scan rate of 0.1 mV s^−1^ vs. Li^+^/Li. It is well known from the previous studies that CV curves of the S/DPAN system are different from that of the S/C composite electrodes due to an interaction of sulfur with PAN [52]. A broad reduction peak was observed at around 1.2 V vs. Li^+^/Li in the first CV cycle of the S/DPAN/CNT composite, which shifted to the higher potentials upon the following cycles (Figure 5a). As mentioned in previous studies, the first discharge of S/PAN systems is irreversible and the reduction peak could be due to the side reactions related to solid electrolyte interface (SEI) and large polarization [53,54]. From the second cycle the system shows two reversible overlapping reduction peaks at around 1.9 and 1.6 V and a broad oxidation peak at around 2.4 V. The first small reduction peak at 1.9 V was related to formation of high order polysulfides and the second one at 1.6 V was attributed to further reduction of polysulfides into lithium sulfide [55]. Figure 5b shows the potential profiles of the S/DPAN/CNT electrodes on the cPAN CNF current collector with 1.2 mg cm^−2^ sulfur loading at a current density of 0.1 C. The S/DPAN/CNT electrode shows a large initial discharge capacity of 2000 mAh g^−1^, which is typical for the S/DPAN system [56]. The composite cathode delivered a high discharge capacity of 1620 mAh g^−1^ within the second cycle and gradually reduced to 1208 mA h g^−1^ in the first 50 cycles (Figure 5c). A stable capacity of about 1100 mAh g^−1^ was received at the 100th cycle. The same composite electrode on the Al foil with the similar mass loading offered only 1350 mAh g^−1^ and 870 mAh g^−1^ capacity at the second and 100^th^ cycles (Figure S2). Taking in account that the areal density of whole electrode including the cPAN current collector was 4.06 mg cm^−2^, which was lighter than the electrode on the Al foil with the areal density of 8.10 mg cm^−2^ the capacity was calculated for the unit mass of electrode as 200 and 500 mAh g^−1^, respectively. So, the capacity of the electrode was increased 2.5 times by replacing the Al foil with an ultralight and porous cPAN CNF current collector. It is one of the best results so far reported for the sulfur composite cathode of the 3D carbon current collector, e.g., the S/C composite cathode on commercial carbon fiber paper with a capacity of 1278 mAh g^−1^ (calculated per sulfur) and ~300 mAh g^−1^ (calculated per electrode) [27]. The coulombic efficiency of the composite cathode was about 100% over cycling, which could be related with the successfully suppression of polysulfides shuttle effect by the S/DPAN system and 3D structure of CNFs [57].

**Figure 6:**
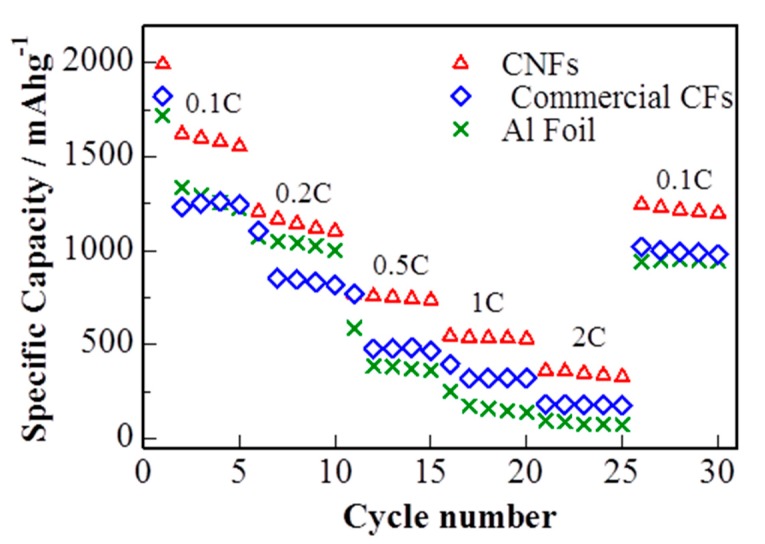
Rate capability of cells with sulfur composite cathode on cPAN CNF, commercial CNF current collector and Al foil (discharge capacity).

The rate capability of the S/DPAN/CNT electrodes with sulfur loading of 1.2 mg cm^−2^ at different current rates (0.1 C–2 C) is shown in Figure 6. The reversible discharge capacities are 1207, 770, 544 and 360 mAh g^−1^ at constant current rates of 0.2 C, 0.5 C, 1 C and 2 C, respectively. While the current density was reduced back to 0.1 C, the discharge capacity of the electrode recovered to 1250 mAh g^−1^, showing a good tolerance toward a high electric current impact. S/DPAN/CNT composite cathode on cPAN CNF overperformed the electrode on commercial CF and Al foil with the same loading of active material. S/DPAN/CNT cathode on commercial CF offered only 1260 mAh g^−1^ at 0.1 C and dropped to 141 mAh g^−1^ at 2 C, while the cathode on Al foil failed at the current density higher than 0.5 C.

The better C rate performance of the cathode on cPAN can be explained by improved bulk conductivity owing to its 3D nanoarchitecture. The relatively poor rate capability of the same electrode on commercial CF in comparison with cPAN CNF current collector is related to lower bulk conductivity and weak adhesion of S/DPAN/CNT particles on fibers. As it is seen from SEM images, the diameter and void distance between fibers of commercial CF were larger for one order and incapable to hold the composite electrode particles (Figure S3), which resulted in lower electrical conductivity. The comparison of charge/discharge profiles in Figure 5a, Figure S2a,c show that the lowest polarization was observed for the cathode on cPAN CNF and the highest for Al foil, which correlated with the trend of C rate performance.

**Figure 7:**
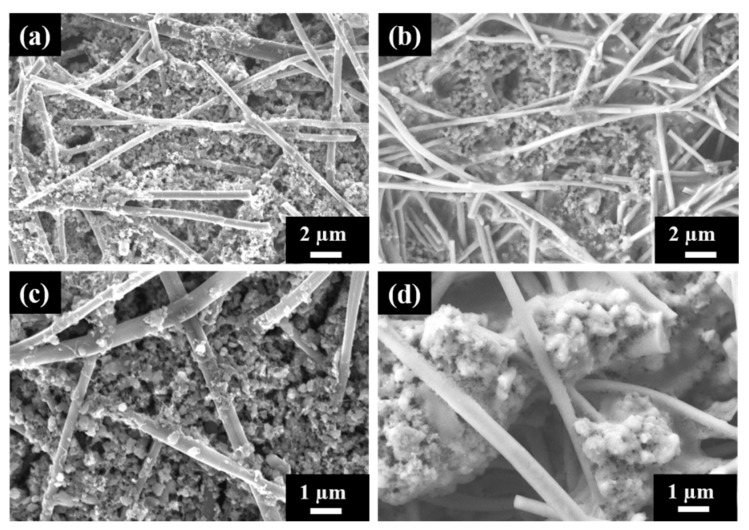
SEM images of S/DPAN/CNT composite cathode on the cPAN CNF current collector (**a**,**c**) fresh and (**b**,**d**) after 100 cycles of discharge.

The morphology of the electrode with cPAN CNFs loaded with the sulfur composite cathode was analyzed using SEM before and after 100 cycles of charge–discharge as shown in Figure 7. Figure 7b,d shows the surface morphology of the cycled electrode in two different magnification and also in comparison with the fresh one (Figure 7a,c). The S/DPAN/CNT electrode exhibited just a little change in surface morphology during the cycling test and the stability and adherence of composite cathode within the nanofibers were very well maintained. The composite cathode particles became denser and agglomeration was observed after cycling. This could be related with PVdF binder used in cathode preparation, which led to the agglomeration of the irreversible Li_2_S_2_/Li_2_S phase with increasing cycles as explained in literature [58]. The existing void space between nanofibers provides extra space for intermediate products and inhibit the shuttle effect during reaction as mentioned above in the electrochemical performance test results [59].

## 4. Conclusions

Carbon nanofibers were successfully prepared with the controllable diameters less than 2 µm by the simple electrospinning method. Optimal conditions of carbonization were established for PAN nanofibers. The ultralight and porous cPAN CNFs could accommodate a large amount of the S/DPAN/CNT composite cathode resulting in a sulfur mass loading of around 1.2 mg cm^−2^. Owing to its unique structure, cPAN NFs remarkably improved the cycle performance and rate capability of the electrode compared to the one on the Al foil. cPAN NFs are lightweight, chemically stable and have nanosized voids and fibers, which ensure the compact packing of active material and provide high bulk conductivity of the electrode. Therefore, the capacity of the electrode was increased 2.5 times by replacing the Al foil with an ultralight and porous cPAN CNF current collector. Additionally, they might help to suppress the polysulfide dissolution by trapping them into their porous structure, thus the developed system could be a promising candidate for high performance Li/S batteries.

## Supplementary Materials

The following are available online at https://www.mdpi.com/2079-4991/10/4/745/s1, Figure S1: SEM images of (a) stabilized at 280 °C, carbonized at (b) 600 °C and (c) 700 °C PAN12 nanofibers, Figure S2: (a, b) Potential profile and cycle performance of sulfur composite on the Al foil, and (c, d) on commercial CFs at 0.1 C, respectively, Figure S3: SEM image of sulfur based composite cathode on commercial CF current collector, Table S1: Diameter and void size of prepared nanofibers.
