# Supplementary material for: Electrospun 3D Structured Carbon Current Collector for Li/S Batteries
Source: Nanomaterials (Basel). 2020 Apr 14;10(4):745. doi: 10.3390/nano10040745 (PMC7221739; doi:10.3390/nano10040745)
Supplement: Supplementary file 1 [file nanomaterials-10-00745-s001.pdf]

Supplementary Materials:

Electrospun 3D Structured Carbon Current Collector for Li/S Batteries

Sandugash Kalybekkyzy[1, 2, 3], Almagul Mentbayeva[1,2,*], Yerkezhan Yerkinbekova[1], Nurzhan Baikalov[2], Memet Vezir Kahraman[3] and Zhumabay Bakenov[1,2]

1 National Laboratory Astana, Nazarbayev University, Institute of Batteries, Nur-Sultan 010000, Kazakhstan; sandugash.kalybekkyzy@nu.edu.kz (S.K.); yerkezhan.yerkinbekova@nu.edu.kz (Y.Y.)

2 School of Engineering and Digital Sciences, Nazarbayev University, Nur-Sultan 010000, Kazakhstan; almagul.mentbayeva@nu.edu.kz (A.M.); nurzhan.baikalov@nu.edu.kz (N.B.); zbakenov@nu.edu.kz (Z.B.)

3 Department of Chemistry, Marmara University, Istanbul 34722, Turkey; mvezir@marmara.edu.tr (M.V.K.)

* Correspondence: almagul.mentbayeva@nu.edu.kz; Tel.: +7-777-395-4749

Received: 12 February 2020; Accepted: 9 March 2020; Published: date

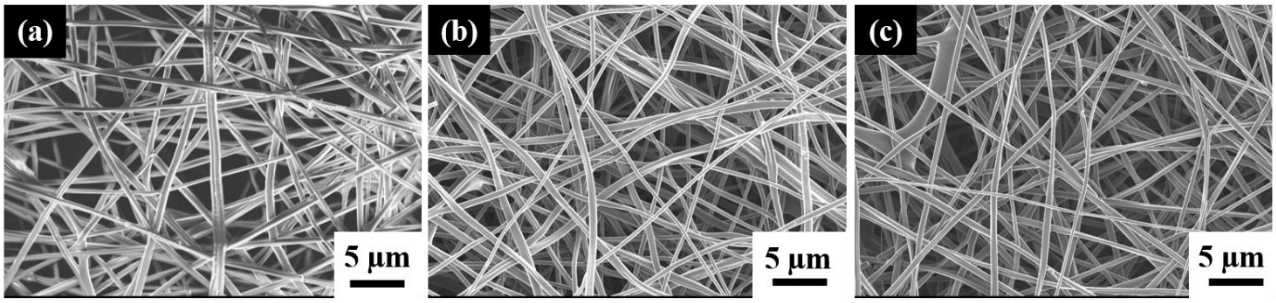

Figure S1. SEM images of (a) stabilized at 280 °C, carbonized at (b) 600 °C and (c) 700 °C PAN12 nanofibers.

Table S1. Diameter and void size of prepared nanofibers.

**Name**	**Diameter of fiber, nm**	**Void distance, µm**	**Areal density, mg cm^{-2}**	**Thickness, µm**
PAN10	~500	-		
PAN12	~1300	-		
PAN14	~2500	-		
cPAN10	~300	≤ 1	0.85	
cPAN12	~800	≤ 1.5		~25
cPAN14	~1500	≤ 3		
Commercial carbon fibers	~7000	≤ 20	6.03	
S/DPAN/CNT particle size ≤ 1.5 µm				
Al current collector			4.9	~15

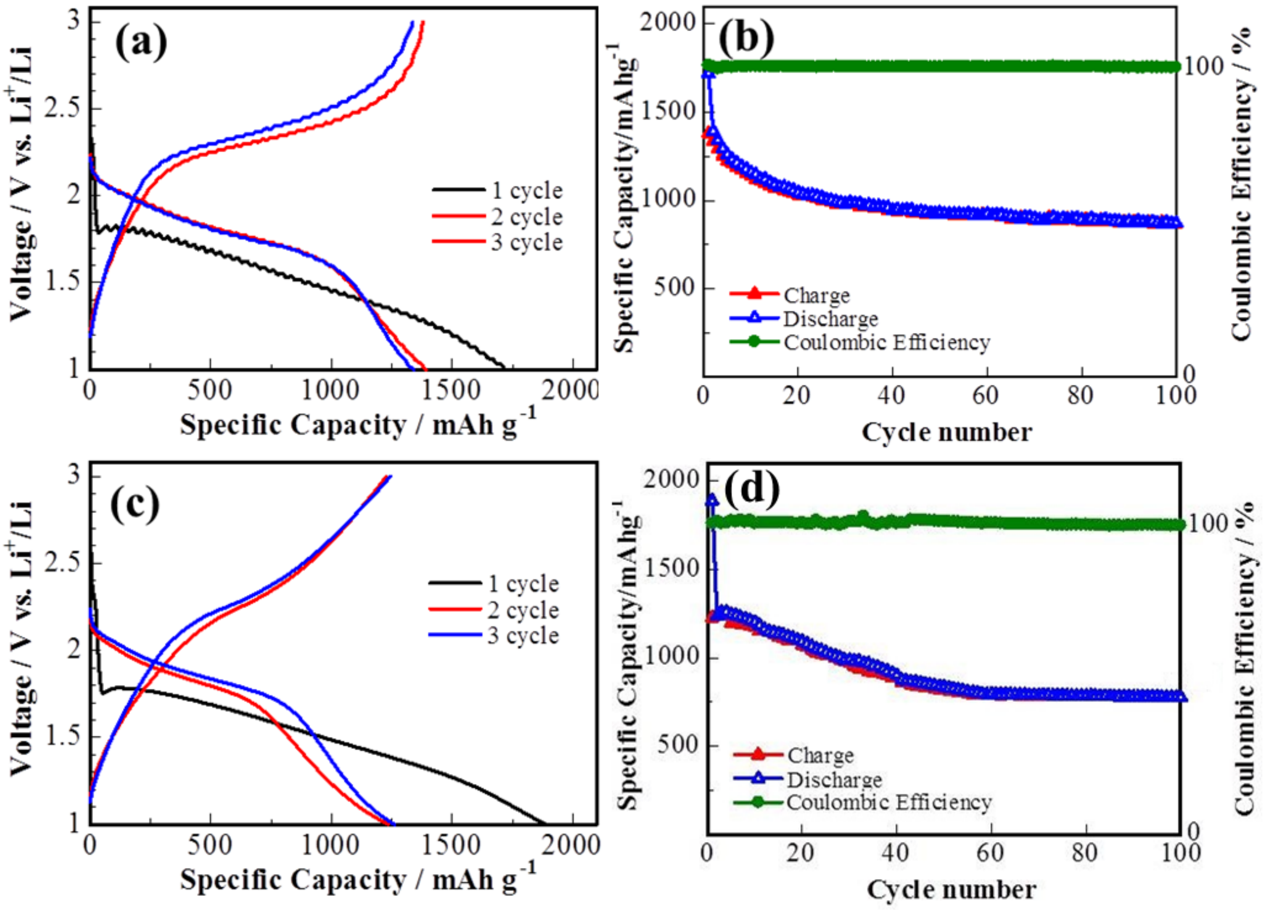

Figure S2. (**a**, **b**) Potential profile and cycle performance of sulfur composite on Al foil, and (**c**, **d**) on commercial CFs at 0.1 C, respectively.

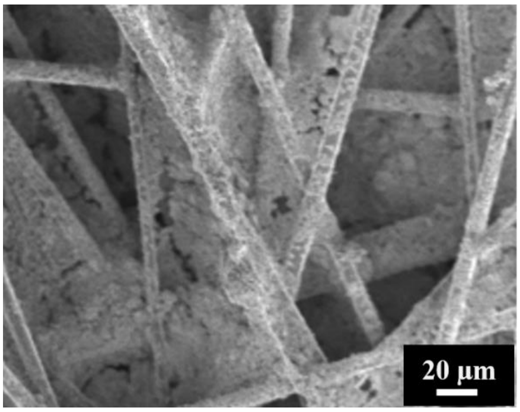

Figure S3. SEM image of sulfur based composite cathode on commercial CF current collector.
